# Outcome and diagnostic reproducibility of the thyroid cytology “indeterminate categories” SIAPEC/SIE 2014 in a consecutive series of 302 cases

**DOI:** 10.1007/s40618-020-01377-4

**Published:** 2020-08-14

**Authors:** F. Massa, P. Caraci, A. Sapino, G. De Rosa, M. Volante, M. Papotti

**Affiliations:** 1 Department of Oncology, University of Turin and Pathology Unit, San Luigi Hospital, Regione Gonzole 10, 10043 Orbassano, Torino Italy; 2 Internal Medicine Unit, San Luigi Hospital, Orbassano, Turin Italy; 3 Department of Medical Sciences, University of Turin, Turin, Italy; 4 Candiolo Cancer Institute, FPO-IRCCS, Candiolo, Turin Italy; 5 Pathology Unit, Mauriziano Hospital, Turin, Italy; 6 Department of Oncology, University of Turin, and Pathology Unit, “Città della Salute e della Scienza” Hospital, Turin, Italy

**Keywords:** Thyroid cytology, Indeterminate lesions, Classification, Risk of malignancy, Inter-observer agreement

## Abstract

**Purpose:**

The clinical impact of the SIAPEC/SIE 2014 classification for thyroid cytology has been addressed in few studies that evaluated the malignancy rate and the relative prevalence of each category. No study analyzed its intra-observer and inter-observer reproducibility, so far.

**Methods:**

We retrospectively collected all “indeterminate” lesions diagnosed before (2011–2014) and after (2015–2018) the application of the SIAPEC/SIE 2014 classification at our Institution. Their relative malignancy risks were calculated based on available histological diagnoses. Cytological and clinical features of TIR3A were compared with the surgical outcome. Finally, a large set of samples was re-evaluated in blind of the original cytological and histological diagnoses by two pathologists, independently.

**Results:**

The prevalence of “indeterminate” diagnoses increased in years 2015–2018 (302/1482, 21% with 14% of TIR3A and 7% TIR3B categories) compared to years 2011–2014 (261/1680, 16%). Surgery was performed in 27% TIR3A and in 97% TIR3B cases. Malignancy rates were 40% for TIR3B and 17% for TIR3A, but were greatly influenced by the adoption of the WHO 2017 re-classification of encapsulated follicular-patterned lesions (decreasing to 28% and 6%, respectively). No criteria except for tumor size were associated to malignancy in TIR3A category. Intra-observer agreement of the experienced pathologist was 122/141 (86%), whereas inter-observer agreement between the expert and in-training pathologist was 95/141 (67%).

**Conclusions:**

In this real-life experience, the sub-classification of TIR3A and TIR3B slightly increased the overall prevalence of “indeterminate” diagnoses. Malignancy rates were higher than estimated for both TIR3A and TIR3B categories. Agreement among observers highly depended on pathologist’s training.

## Introduction

Thyroid nodules are diagnosed with increasing frequency in clinical practice in the general population [1, 2]. This phenomenon is due to both an increased exposure to risk factors and to an improvement in diagnostic tools [3]. Despite the high number of subjects with thyroid nodules, the prevalence of cancer is low. For this reason, the first step in the management of patients is an accurate triage of those who should undergo surgery, based on the complementary evaluation of anamnestic, clinical, and ultrasound information.

Cytology is a major diagnostic tool, because is safe, cheap, minimally invasive, and generally accurate in distinguishing most benign and malignant nodules [4]. However, indeterminate diagnostic categories (inconclusive for malignancy) represent 20–25% of cytological diagnoses, with only a small part of these eventually being a cancer. Therefore, a pre-operative stratification of indeterminate diagnoses into low and high risks for malignancy is a major challenge for clinicians and cytopathologists [5]. The two major classification systems currently used worldwide, the Bethesda Reporting System for Thyroid Cytology (BRSTC) and the British Thyroid Association Classification (UK RCPath), divide the indeterminate category into two sub-groups. In particular, BRSTC describes “atypia of undetermined significance (AUS)/follicular lesion of undetermined significance (FLUS)” and “follicular neoplasm (FN)/suspicious for FN (SFN)” [6], while UK RCpath distinguishes “Thy3a” for atypia and “Thy3f” for follicular neoplasm [7]. In 2014, the Italian Society of Anatomic Pathology and Cytology (SIAPEC) together with the Italian Society of Endocrinology (SIE) published a new classification system based on six categories, in line with the other two systems, identifying within the indeterminate cytological category a low-risk indeterminate lesion (TIR3A) and a high-risk indeterminate lesion (TIR3B) [8]. Such categories have different expected rates of malignancy (< 10% for TIR3A and 15–30% for TIR3B) [8], but, in the SIAPEC/SIE, as well as for the other classification systems, they have a high degree of overlap of some cytological features that potentially mine their diagnostic accuracy [9]. Moreover, estimated rates of malignancy in the 2014 SIAPEC/SIE classification are only partially based on the published data at the time of their publication and thereafter. Finally, the diagnostic reproducibility of the SIAPEC/SIE 2014 classification has never been investigated, so far.

The pre-operative molecular characterization of thyroid nodules through the use of different approaches has been proposed as a complement to cytopathology to refine malignancy risk in thyroid cytology. In the last decades, thanks also to the Thyroid Cancer Genome Atlas [10], the number of tumors with unknown genetic drivers has been considerably reduced. Molecular tests might be either performed focusing on a restricted panel of genes, selecting the most prevalent or those associated with adverse outcome [11], or following the advent of next -generation sequencing technology, using expanded panels, such as the ThyroSeq® panel whose third generation recently obtained clinical validation [12]. Additional approaches to estimate the risk of malignancy in thyroid FNAs include microRNA analysis, which is a promising tool [13], but still needs independent validation for entering the clinical practice. National and International Guidelines [4, 14] recommend ancillary molecular analysis if accessible, especially for indeterminate categories, but their costs, with special reference to multi-gene panels, make their accessibility still limited. The most cost-effective strategy is probably to tailor thyroid cancer-specific panels [15] that decrease the overall test costs, but have a major problem in terms of standardization and methodological reproducibility.

The present retrospective study aimed at evaluating the impact of the 2014 SIAPEC/SIE classification on the prevalence of each individual category, at calculating the factual malignancy rate of the low-risk and high-risk indeterminate categories and at testing their inter-observer and intra-observer reproducibility.

## Materials and methods

All cytological diagnoses performed at the Pathology Unit of the San Luigi Hospital, Orbassano, Turin from February, 1st 2011 to December, 31st 2018 were retrospectively considered. All patients underwent an endocrinological counseling, a neck ultrasound examination, and a fine needle aspiration (FNA) of the most significant nodule(s). For all indeterminate cytological diagnoses, clinical and ultrasound characteristics (laterality, size, echogenicity, halo, margins, calcifications, and vascularization) were retrieved. All FNA was performed with a sterile 20 ml syringe and a 22Gauge needle by a single experienced endocrinologist (PC) under the guidance of ultrasound with on-site assistance by a pathologist to assess adequacy. Available material for each sample included from 2 to 6 smears (hematoxylin and eosin and Giemsa stained) and hematoxylin and eosin slides from cell block preparations.

All cytological diagnoses were divided into those performed before and after February, 1st 2015, that represents at our institution the date of shift from the 2007 to the 2014 SIAPEC/SIE classifications. For all indeterminate lesions diagnosed with the SIAPEC/SIE 2014 classification, histological reports of surgically resected cases were retrieved from the Pathology files of four major hospitals in the Turin area dedicated to thyroid surgery (San Luigi Hospital, Città della Salute e della Scienza Hospital, Mauriziano Hospital, and FPO-IRCCS of Candiolo) and the malignancy rate for both TIR3A and TIR3B was assessed. Since TIR3A has an indication to repeat FNA, the concordance between the initial diagnosis and the repetitive cytological diagnosis on the same thyroid nodule was also recorded. Reproducibility was assessed evaluating all consecutive indeterminate diagnoses (both TIR3A and TIR3B) rendered in years 2015 and 2016, blind of the original cytological diagnosis and surgical outcome, whenever available. To assess intra-observer agreement, all cases were reviewed by the experienced thyroid pathologist (MV) who was also responsible for the original diagnoses, whereas for inter-observer agreement, the same series was also independently evaluated by a senior resident in pathology (FM).

The Chi-square test or Fisher’s exact test, as appropriate, were used to compare ultrasound and clinical features with surgery and histological outcome. Student’s t test was used to compare continuous variables. All analyses were performed using GraphPad software (Graphpad Software Inc., La Jolla, CA). All *p* values of 0.05 or less were considered statistically significant.

## Results

The adoption of the SIAPEC/SIE 2014 classification had an impact on the overall prevalence of “indeterminate” diagnoses. In fact, TIR3 diagnoses in years 2011–2014 (up to February 1st, 2015) were 261/1680 (16%), whereas subsequently increased up to 302/1482 (21%) overall, including 207 TIR3A (14%) and 95 TIR3B (7%). Such an increase was associated with a decrease of the benign TIR2 category (from 896/1680, 53%, to 715/1482, 48%), whereas the prevalence of all other categories remained stable (Table 1). No significant clinical differences were observed between TIR3A and TIR3B cases (Table 2). Predominant oncocytic features were observed in 32/207 (15%) TIR3A and 13/95 (14%) TIR3B cases. Regarding follow-up, we found information for 175/207 (84%) TIR3A and for 89/95 (94%) TIR3B patients.

**Table 1:** Distribution of diagnostic category before and after the application of the SIAPEC/SIE 2014 system

Diagnostic category	2011/2014^a^, #1680 (%)	2015/2018, #1482 (%)
TIR1	432 (26)	385 (26)
TIR2	896 (53)	715 (48)
TIR3	261 (16)	302 (21) TIR3A: 207 (14) TIR3B: 95 (7)
TIR4	32 (2)	32 (2)
TIR5	59 (3)	48 (3)

^a^Up to February 1st 2015.

**Table 2:** Major features of TIR3A and TIR3B cases

Parameter	TIR3A, #207	TIR3B, #95
Age, median (range)	56 (18–85)	51 (18–84)
M/F (ratio)	59/148 (1/2.5)	31/64 (1/2.1)
Multinodularity (%)	123 (59)	57 (60)
Size in mm, mean (range)	24 (8–80)	25 (10–70)
Predominant oncocytic features (%)	32 (15)	13 (14)

A second cytological sample was obtained in 26 TIR3A cases, with a concordant diagnosis in 13/26 (50%), the other diagnoses being TIR3B in 3, TIR2 in 6, and TIR1 in 4 cases. The three cases re-classified as TIR3B underwent surgery with a final histological diagnosis of NIFTP, FT-UMP, and goiter, respectively.

Surgery was performed in 48/175 (27%) TIR3A and in 86/89 (97%) TIR3B cases for which follow-up information was available. This number complies with the recommendations of the SIAPEC/SIE consensus [8]. The three non-operated TIR3B cases had all important contraindications to surgery. In the 175 TIR3A group, we analyzed the differential clinical and ultrasound features between cases that underwent surgery and those with follow-up, only. The mean follow-up of the 127 TIR3A cases not undergoing surgery was 22 months (median 17, range 7–57). Surgically treated cases showed a significant younger age (51 vs 58 years, *p* = 0.005), larger nodule size (28 vs 23 mm, *p* = 0.005), and less common hypoechogenicity of the nodules (18/48 vs 76/127, *p* = 0.01). All other features, including sex, multinodularity, location, “taller than wide” features, halo, calcifications, or vascularization, were not associated with the clinical decision of surgical treatment. Malignancy rate based on the surgical outcome was 40% for TIR3B and 17% for TIR3A. Malignancy rate was greatly influenced when “noninvasive follicular thyroid neoplasm with papillary-like nuclear features” (NIFTP), “follicular tumor with uncertain malignant potential” (FT-UMP), or “well-differentiated tumor with uncertain malignant potential” (WDT-UMP) categories were included into the benign group, decreasing to 28% for TIR3B and 6% for TIR3A (Table 3). The clinical (age, sex, multinodularity, location, and size), ultrasonographic (“taller than wide” features, echogenicity, halo, calcifications, and pattern of vascularization), and cytological (cellularity, cytological atypia, regressive changes, and predominant oncocytic features) characteristics of TIR3A cases with benign vs malignant histological diagnoses were re-assessed. Larger tumor size was the single feature distinctive of TIR3A cases with malignant (NIFTP, WDT-UMP, and FT-UMP included) surgical outcome (38 vs 26 mm, *p* = 0.02).

**Table 3:** Surgical outcome of TIR3A and TIR3B nodules

Histological diagnoses	TIR3A #48, (%)	TIR3B #86, (%)
Goiter	22 (46)	16 (19)
Follicular adenoma	18 (37,5)	36 (42)
NIFTP^a^	1 (2)	4 (5)
FT-UMP^b^	3 (6)	1 (1)
WDT-UMP^c^	1 (2)	5 (6)
Follicular carcinoma	2 (4.5)	2 (2)
Papillary carcinoma	1 (2)	21 (24)
Poorly differentiated carcinoma	0	1 (1)
Malignancy rate (with NIFTP^a^, FT-UMP^b^ and WDT-UMP^c^)	17%	40%
Malignancy rate (without NIFTP^a^, FT-UMP^b^ and WDT-UMP^c^)	6%	28%

^a^Noninvasive follicular thyroid neoplasm with papillary-like nuclear features

^b^Follicular tumor with uncertain malignant potential

^c^Well-differentiated tumor with uncertain malignant potential

**Fig. 1:**
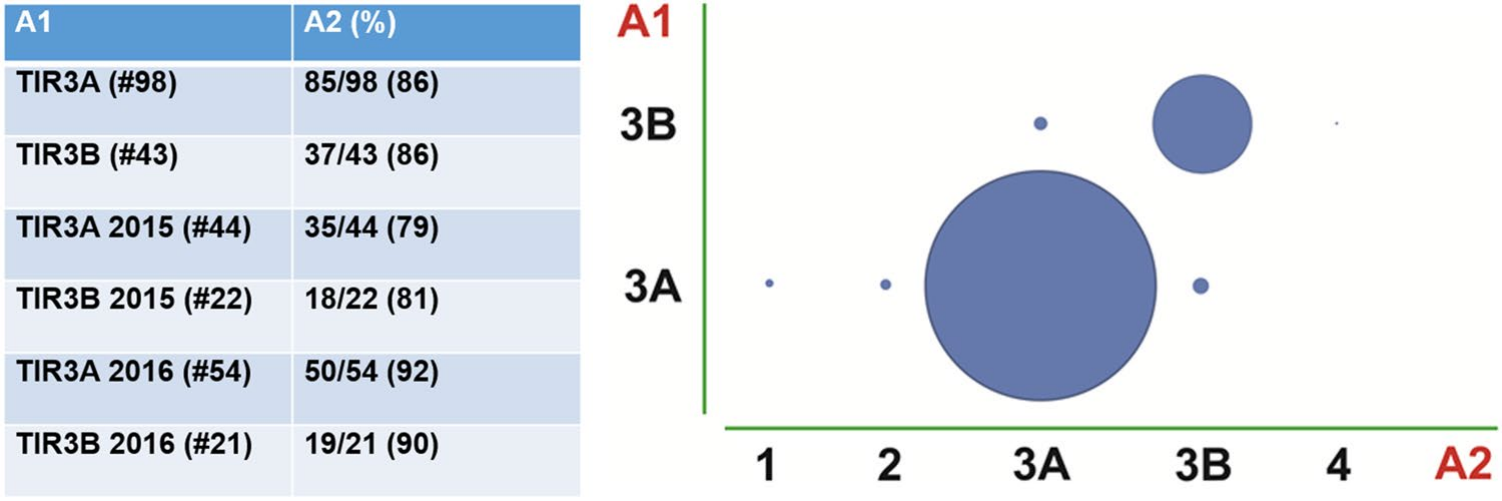
Intra-observer agreement in indeterminate lesions comparing original diagnosis (A1) and re-evaluation (A2) from the same experienced pathologist

**Fig. 2:**
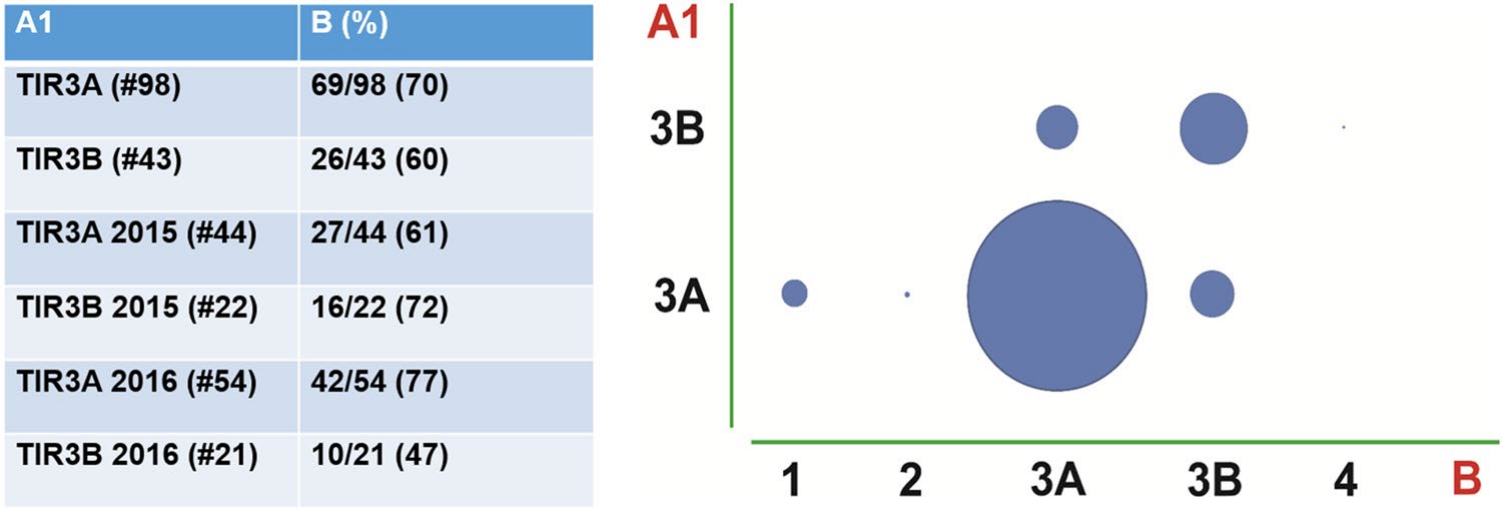
Inter-observer agreement in indeterminate lesions comparing original diagnosis (A1) and re-evaluation (B) from a pathologist in training

Diagnostic reproducibility was tested in 144 TIR3A and TIR3B consecutive cases from 2015 to 2016. Three samples were subsequently excluded for the poor quality of the available material (faded or dismounted smears). Intra-observer overall agreement (same pathologist for original diagnosis and re-evaluation) was 86% (122/141 concordant diagnoses), equal in both TIR3A (85/98 concordant diagnoses) and TIR3B (37/43 concordant diagnoses) sub-groups (Fig. 1). In addition, the intra-observer agreement raised from 53/66 (80%) to 69/75 (92%) comparing diagnoses of year 2015 and year 2016, respectively. Inter-observer agreement between expert (original diagnosis) and in-training pathologist was lower, with an overall concordance of 67% (95/141 concordant cases), ranging from 70% (69/98 concordant cases) for TIR3A to 60% (26/43 concordant cases) for TIR3B cases (Fig. 2). Discordant diagnoses mostly included TIR3A versus TIR3B and vice versa, with a minority of cases being re-classified into other categories (including TIR1, TIR2, and TIR4). The overall agreement was randomly distributed comparing cases originally diagnosed in years 2015 and 2016.

## Discussion

The SIAPEC/SIE 2014 classification for thyroid cytology introduced, among others, a major modification of the indeterminate TIR3 category, as coded in the previous proposal. This modification was intended to guide clinicians when facing with an indeterminate lesion, supporting two major strategies: follow-up for the newly coded low-risk indeterminate lesion TIR3A and surgery for the high-risk indeterminate lesion TIR3B. However, after the publication of this new classification, a few studies (and some from the same group) aimed at verifying the impact of this classification in the real life. We, therefore, designed the present study to assess in a single center and in a large time frame the clinical and pathological impact of the application of the new SIAPEC/SIE 2014 classification. Our data are clean from any methodological bias or any heterogeneity of clinical management, since nearly all patients were followed by the same clinician performing FNA and cytological diagnoses were performed by a single pathologist. Moreover, all cytological samples were prepared using a single methodology.

A first impact of the SIAPEC/SIE 2014 classification was an increase of the overall TIR3 indeterminate diagnoses along years. In fact, an increase from 16 to 21% was observed comparing two similar time-frames and comparable number of overall thyroid cytology diagnoses, before and after the adoption of the SIAPEC/SIE 2014 classification. While the prevalence of all other categories remained stable, a decrease in TIR2 diagnosis (from 53 to 48%) was evident, an observation very similar to what reported by Sparano et al. [10] and suggesting that some nodules previously labeled as TIR2 were prudently classified as TIR3A using the new classification.

The observed malignancy rates for TIR3A and TIR3B were 17% and 40%, respectively, values that are pretty much higher than those estimated in the classification scheme (< 10% for TIR3A and 15–30% for TIR3B) [8]. With all the limitations due to the relatively small sample size of cases with cytological/histological correlation in our study, the percentages observed in our series are in line with available data, both from the experience of individual groups [16–25] and from meta-analyses [26, 27]. Although these data confirm that the sub-classification into low- and high-risk groups identifies two significantly different patient populations with a relevant clinical impact, they also claim a potential underestimation of the risk of malignancy in the TIR3A category whose major clinical indication is follow-up. A major potential bias in this setting might be represented by the fact that TIR3A cases undergoing surgery are selected based on additional “high risk for malignancy” clinical features. However, comparing the major characteristics of TIR3A cases with surgical treatment *vs* those with follow-up, we could not identify any difference except for younger age and larger size in the former group. Indeed, hypoechogenicity (an ultrasonographic characteristic of suspicion) was more frequently detected in the follow-up group. Therefore, our data suggest that the two groups are not that different and so it might be their malignancy risk. Moreover, a larger size was the single parameter different in TIR3A cases with histological diagnosis of malignancy as compared to those histologically benign. In this specific context, the relatively short follow-up of our series might not be informative enough to really depict the clinical outcome of TIR3A cases, and further prospective observations are needed.

By contrast, our data on the malignancy risk for TIR3B are more robust, since almost all cases underwent surgery, and reinforce the concept that the malignancy risk in the original SIAPEC/SIE 2014 proposal was underestimated. In fact, our data are comparable to the recent study by Sparano et al. [16] (40.4%) but still lower than those reported in the meta-analysis by Trimboli et al. [26] (52%) evaluating six studies with a malignancy rate ranging from 42 to 57% [17–22]. A recent study also reported a malignancy rate of 38.5% in TIR3B cases, claiming that age < 55 years, size < 20 mm, and presence of microcalcifications and of some nuclear features suggestive of papillary thyroid carcinoma further define a subset of cases with increased risk of malignancy [28]. All the above results strongly suggest that population selection bias even at national level may influence the relative malignancy rate by category, as observed in a worldwide setting [29].

Another relevant issue is what has to be included in the “malignancy” category, when evaluating the outcome of surgically resected nodules. In fact, the new 2017 WHO classification of thyroid tumors [30] incorporated the novel concept of a category of follicular neoplasms whose clinical behavior is indolent despite equivocal nuclear features for papillary carcinoma or questionable features of capsular invasion. When we re-analyzed the malignancy rate by putting cases with uncertain malignant potential (FT-UMP and WDT-UMP) and NIFTP in the benign group, the results strongly changed, being reduced to 28% for TIR3B and 6% for TIR3A. The impact of this new terminology in the risk of malignancy calculation for indeterminate categories is a matter of debate, irrespective of the used classification system for reporting thyroid cytology [27, 31]. However, it is important to consider that, although these diagnoses are considered clinically indolent, they require appropriate surgery for their treatment and ultimate diagnostic classification.

The final aim of our study was to evaluate the reproducibility of the indeterminate categories of the new system SIAPEC/SIE 2014 between two pathologists with different professional experience. Our data underline a relatively low reproducibility between the expert pathologist and the in-training pathologist. Discordant diagnoses mostly included TIR3A versus TIR3B and vice versa, whereas a minority of cases were re-classified into other categories including TIR1, as a proof that paucity of the material available for diagnosis represents a limiting factor in the correct application of the classification. As a matter of fact, comparing the agreement between observers for “indeterminate categories” in the few studies available in the literature for the BRSTC [32, 33] and UK RCPath [34], the results for the SIAPEC/SIE 2014 are apparently much more comforting. However, since the diagnostic criteria for individual classes—with special reference to “indeterminate” categories—are not completely overlapping in SIAPEC/SIE, BRSTC, and UK RCPath systems, as also stated in the original SIAPEC/SIE consensus paper [8], our reproducibility data cannot be directly compared with the Literature. Moreover, our data are obtained between two observers, only, from the same single Institution and with no heterogeneity of sample preparation. The intra-observer reproducibility was much higher and showed an improvement comparing those cases in the first year after adoption of the new classification and in the second year, as a consequence of a more confident and homogeneous application of the system. The impact of training in diagnostic reproducibility applying the BRSTC system was already tested by Pathak and coworkers comparing a consultant pathologist vs a senior and a junior residents [35]. However, our results include also intra-observer reproducibility, and, altogether, indicate that improving training and experience is a major goal to reach the best performance in terms of concordance and overall diagnostic accuracy of this classification.

In conclusion, in the present study, we show that (i) the new 2014 SIAPEC/SIE sub-classification of TIR3A and TIR3B split low risk and high risk of malignancy “intermediate cases” fairly well, though with some heterogeneity and with a risk of malignancy higher than that estimated for both categories, (ii) the agreement among observers for TIR3A and TIR3B classes highly depends on pathologist’s training and familiarity with newly proposed cytological parameters, and (iii) the recent histological intermediate entities of follicular tumors with uncertain malignant potential and/or non-invasive features, drastically reduce the risk of malignancy in such indeterminate cytological categories.
